# Predicting difficult laryngoscopy in morbidly obese Thai patients by ultrasound measurement of distance from skin to epiglottis: a prospective observational study

**DOI:** 10.1186/s12871-022-01685-7

**Published:** 2022-05-14

**Authors:** Sumidtra Prathep, Wilasinee Jitpakdee, Wisara Woraathasin, Maliwan Oofuvong

**Affiliations:** Department of Anesthesiology, Faculty of Medicine, Songklanagarind Hospital, Prince of Songkla University, 15 Kanjanavanich Road, Hat Yai, Songkhla, Songkhla 90110 Thailand

**Keywords:** Ultrasound airway, Difficult laryngoscopy, Difficult airway, Obesity, Prediction

## Abstract

**Background:**

In morbidly obese patients, airway management is challenging since the incidence of difficult intubation is three times than those with a BMI within the healthy range. Standard preoperative airway evaluation may help to predict difficult laryngoscopy. Recent studies have used ultrasonography-measured distance from skin to epiglottis and pretracheal soft tissue at the level of vocal cords, and cut-off points of 27.5 mm and 28 mm respectively have been proposed to predict difficult laryngoscopy. The purpose of this study is to evaluate ultrasonography-measured distance from skin to epiglottis for predicting difficult laryngoscopy in morbidly obese Thai patients.

**Methods:**

This prospective observational study was approved by the Ethics Committee of the Faculty of Medicine, Prince of Songkla University. Data were collected from January 2018 to August 2020. Eighty-eight morbidly obese patients (BMI ≥ 35 kg/m^2^) requiring general anesthesia with endotracheal intubation for elective surgery were enrolled in the Songklanagarind Hospital. Preoperatively, anesthesiologists or nurse anesthetists who were not involved with intubation evaluated and recorded measurements (body mass index, neck circumference, inter incisor distance, sternomental distance, thyromental distance, modified Mallampati scoring, upper lip bite test, and distance from skin to epiglottis by ultrasound. The laryngoscopic view was graded on the Cormack and Lehane scale.

**Results:**

Mean BMI of the eighty-eight patients was 45.3 ± 7.6 kg/m^2^. The incidence of difficult laryngoscopy was 14.8%. Univariate analysis for difficult laryngoscopy indicated differences in thyromental distance, sternomental distance and the distance from skin to epiglottis by ultrasonography. The median (IQR) of thyromental distance in difficult laryngoscopy was 6.5 (6.3, 8.0) cm compared with 7.5(7.0, 8.0) cm in easy laryngoscopy (*p*-value 0.03). The median (IQR) of sternomental distance in difficult laryngoscopy was 16.8 (15.2, 18.0) cm compared with 16.0 (14.5, 16.0) cm in easy laryngoscopy (*p*-value 0.05). The mean distance from skin to epiglottis was 12.2 ± 3.3 mm Mean of distance from skin to epiglottis in difficult laryngoscopy was 12.5 ± 3.3 mm compared with 10.6 ± 2.9 mm in easy laryngoscopy (*p*-value 0.05). Multivariate logistic regression indicated the following factors associated with difficult laryngoscopy: age more than 43 years (A), thyromental distance more than 68 mm(B) and the distance from skin to epiglottis more than 13 mm(C). The scores to predict difficult laryngoscopy was calculated as 8A + 7B + 6C based on the data from our study. One point is given for A if age was more than 43 years old, 1 point is given for B if thyromental distance was less than 6.8 cm and 1 point is given for C if the distance from skin to epiglottis by ultrasonography was more than 13.0 cm. The maximum predicting score is 21, which indicates a probability of difficult laryngoscopy among our patients of 36.36%, odds 0.57, likelihood ratio 3.29 and area under the ROC curve of 0.77, indicative of a good predictive score.

**Conclusions:**

Age, thyromental distance and ultrasonography for the distance from skin to epiglottis can predict difficult laryngoscopy among obese Thai patients. The predictive score indicates the probability of difficult laryngoscopy.

## Introduction

Shiga et al [1] showed that the incidence of difficult intubation in obese patients was three times that of normal weight patients. Obese patients typically have increased amount of adipose tissue deposits in the oral and pharyngeal tissues which decreases the size of the airway and changes the shape of the oropharynx [2]. Furthermore, the patients have short necks with large circumference which contributes to developing airway obstruction and increases the likelihood of difficult direct laryngoscopy for endotracheal intubation under general anesthesia. In addition, obese patients have increased oxygen demand, a decrease in vital capacity, expiratory reserve volume, inspiratory capacity and functional residual capacity, and both low lung compliance and low respiratory system compliance, resulting in the patients being prone to rapid oxygen desaturation and respiratory complication [3–6].

Standard preoperative airway evaluation may help to assess the probability of difficult laryngoscopy. Honarmand et al. [7] reported that the sensitivity of the modified Mallampati score, upper lip bite test, and thyromental distance was 62.5, 48.86 and 37.5%, respectively. Brodsky et al. [8] and Gonazlez et al. [9] showed that difficult intubation is associated with Mallampati score more than 3 and increasing neck circumference. Moreover, Horner et al. [2] found that patients with obstructive sleep apnea syndrome have more fat deposited at the collapsible segment of the pharynx.

The current parameters to assess the difficult airway by using ultrasound were validated in a mainly Caucasian population and that data in a South-East Asian population are lacking. Recent studies have used ultrasonography-measured distance from skin to epiglottis [10] and pre-tracheal soft tissue at the level of vocal cords [11], and cut off values of 27.5 mm and 28 mm respectively were introduced to predict difficult laryngoscopy. The purpose of this study is to predict difficult laryngoscopy in morbidly obese Thai patients by using ultrasonography-measured distance from skin to epiglottis.

## Materials and methods

### Study design

A prospective observational study was approved by the Office of Human Research Ethics Committee, Faculty of Medicine, Prince of Songkla University, Thailand on 17th October 2017 (REC 60–184–08-1), clinicaltrial.gov number TCTR20171226001 on 26th December 2017. Informed consent was obtained from all participants in the study.

### Setting and population

**Fig. 1:**
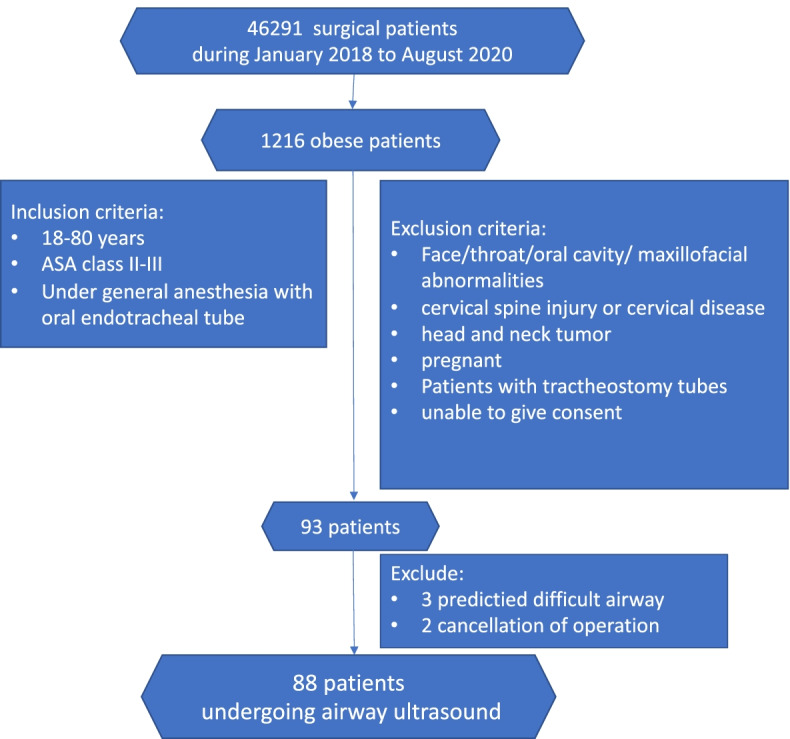
Study Protocol, ASA = American Society Anesthesiologist

The data were collected from January 2018 to August 2020 at Songklanagarind Hospital, Thailand. Inclusion criteria were morbidly obese patient (BMI ≥ 35 kg/m^2^), age between 18 and 80 years, American Society of Anesthesiologists Physical Status (ASA) class II– III, and requiring general anesthesia with endotracheal intubation for elective surgery in Songklanagarind Hospital. Patients were excluded if they had abnormalities of face, throat or oral cavity, maxillofacial abnormalities, cervical spine injury, cervical disease, head and neck tumors. Pregnant patients, tracheostomy patients and patients unable to give consent were excluded as well. The CONSORT diagram is shown as Fig. 1.

### Study protocol

Prior to data collection, two co-investigators were trained to use ultrasound for performing difficult airway clinical screening tests. Airway ultrasound experience was obtained through additional bedside scanning and reviewing journal articles and images.

**Fig. 2:**
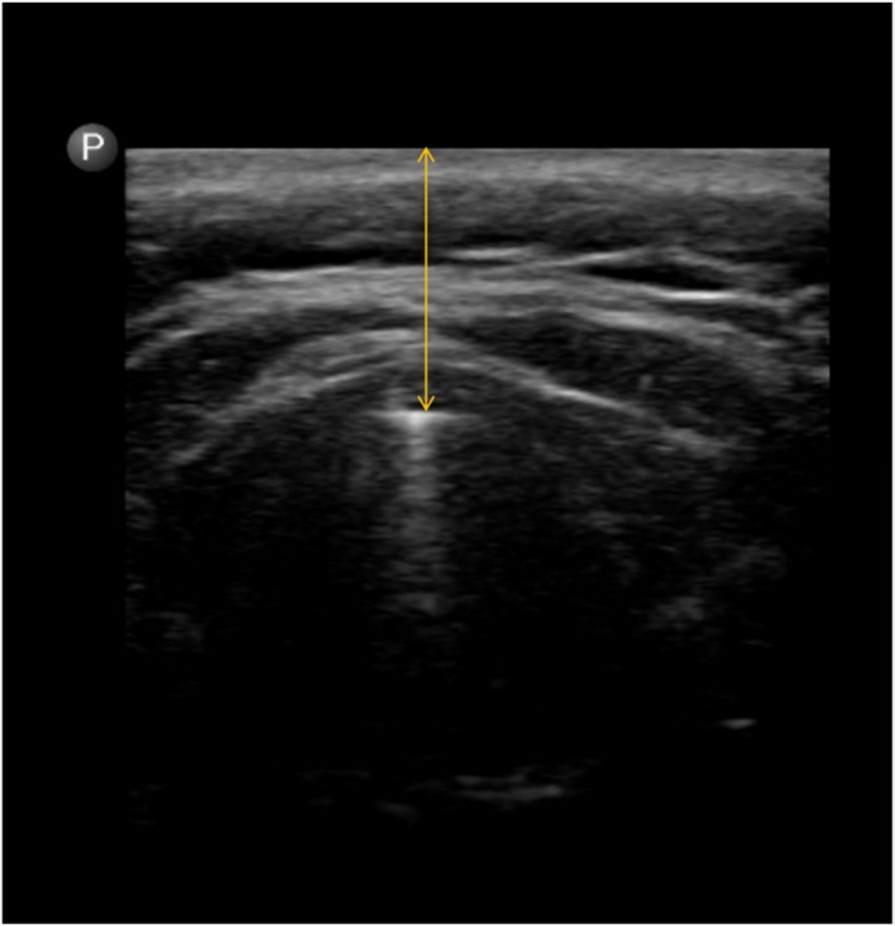
Transverse ultrasound view of the distance between skin and epiglottis at the vocal cord level (arrow)

Preoperatively, anesthesiologists or nurse anesthetists who were not involved in intubation evaluated and recorded measurements (body mass index, neck circumference, inter incisor distance, sternomental distance, thyromental distance, modified Mallampati score, upper lip bite test, and distance from skin to epiglottis by ultrasound) and medical history (snoring, diagnosis of obstructive sleep apnea syndrome). To obtain the ultrasonography-measured distance from skin to epiglottis at the thyrohyoid membrane level in transverse plane (Fig. 2). The participants were placed supine with head and neck in neutral position without a pillow. The Philips Lumify linear array transducer (L12–4,12–4 MHz) was used in this study.

On the day of surgery, the participants were monitored with standard American Society of Anesthesiologists monitors and measurement of end tidal carbon dioxide. Preoxygenation was conducted by administering 100% oxygen via a tight fitting facemask for more than 3 minutes. Attending anesthesiologists could decide the use of any anesthetic agents. The endotracheal intubations were performed using conventional laryngoscope with a McIntosh blade by experienced anesthesiologists who had worked in the operating room for at least 2 years. The grade of laryngoscopic view was recorded by anesthesiologists who performed the endotracheal intubation. The laryngoscopic view was graded on the Cormack and Lehane scale [12] as grade I = full view of the glottis, grade II = partial view of the glottis, grade III = only epiglottis visible, and grade IV = neither epiglottis nor epiglottis visible. The laryngoscopic view, duration of intubation, and number of tracheal intubation attempts were recorded. A Cormack-Lehane grade 1 or 2 was categorized as an easy laryngoscopy, and a grade 3 or 4 was categorized as a difficult laryngoscopy. If the anesthesiologist was unsuccessful within two attempts with conventional Mcintosh laryngoscope, then they proceeded to practice guidelines for management of the difficult airway: an updated report by the American Society of Anesthesiologists Task Force on Management of the Difficult Airway 2013 [13].

Polysomnography was monitored using Apnea-Hypopnea Index (AHI) to test the severity of obstructive sleep apnea (OSA). The classification is grade of mild, moderate and severe sleep apnea. Mild sleep apnea is an AHI of 5–14 events per hour. Moderate sleep apnea is an AHI of 15–29 events per hour. Severe sleep apnea is an AHI of more than 30 events per hour.

### Statistical analysis

The sample size was calculated using this formula for comparison of two independent means:

$${\boldsymbol{n}}_{\mathbf{1}}=\frac{{\left({\boldsymbol{z}}_{\mathbf{1}-\frac{\boldsymbol{\alpha}}{\mathbf{2}}}+{\boldsymbol{z}}_{\mathbf{1}-\boldsymbol{\beta}}\right)}^{\mathbf{2}}\left[{\boldsymbol{\sigma}}_{\mathbf{1}}^{\mathbf{2}}+\frac{{\boldsymbol{\sigma}}_{\mathbf{2}}^{\mathbf{2}}}{\boldsymbol{r}}\right]}{{\mathbf{\Delta }}^{\mathbf{2}}}$$

$$\boldsymbol{r}=\frac{{\boldsymbol{n}}_{\mathbf{2}}}{{\boldsymbol{n}}_{\mathbf{1}}}$$

$$\boldsymbol{\Delta }={\boldsymbol{\mu}}_{\mathbf{1}}-{\boldsymbol{\mu}}_{\mathbf{2}}$$

$${n}_{sen}=\frac{{z^2}_{\frac{\alpha }{2}}\ P\left(1-P\right)}{d^2}$$

$${n}_{total}=\frac{n_{sen}}{incidence}$$

This study used the referent data reported by Ezri T et al. [11] to calculate simple size.

As the incidence of laryngoscopic view grade 3, 4 in obese patient was 22%.

$$\frac{n_{sen}}{0.22}=\frac{19}{0.22}=87$$

The number of patients required for this study was 87.

Statistical analysis was performed using the STATA software. The Shapiro-Wilk normality test was used to assess the normality of continuous variables. Continuous variables were presented as mean and standard deviation (SD). Categorical variables were presented as number of patients and percentages. Continuous variables were analyzed by t-test or Wilcoxon rank sum test. Categorical variables were compared using Fisher’s exact test or Pearson’s Chi-square test. *P*-value less than 0.05 was considered as statistically significant. Kernel density estimation was used to explore the probability distribution of potential predictors of easy laryngoscopy and difficult laryngoscopy. Logistic regression was used to identify airway parameters associated with difficult laryngoscopy. From the best-fitting but parsimonious model, a predictive difficult laryngoscopy score was constructed. An integer weighting score was allocated to each predictive variable in the model such that the ratios among the scores were close to the ratios among the logistic coefficients. The summation of these weighting scores in each patient was used as the predictive score for difficult laryngoscopy. A ROC curve was constructed and used to determine suitable cut-off points that could be used to reflect the different probabilities of having difficult intubation.

## Results

Ninety-three patients were enrolled into the study from January 2018 to August 2020. Three patients were excluded because attending anesthesiologists predicted they may have difficult endotracheal intubation. In addition, two patients were excluded owing to cancellation of the operation. Hence, data from eighty-eight patients were available for the analysis. Patient demographic data are shown in Table 1. BMI (mean ± SD) of the eighty-eight patients was 45.3 ± 7.6 kg/m^2^(minimum BMI = 35.09 kg/m^2^, maximum BMI = 69.79 kg/m^2^).

**Table 1:** Demographic data shown as Number (%), Mean ± SD or Median (IQR)

Variables	Number (%), Mean ± SD or Median (IQR)
Gender	
Male	26 (29.5)
Female	62 (70.5)
Age (year)	43 (29,57)
ASA classification:	
II	2 (2.3)
III	86 (97.7)
BMI (kg/m^2^)	45.3 ± 7.6
Snoring	84 (95.5)
Polysomnography	57 (64.8)
Apnea-Hyponea Index (AHI)	
Mild	11 (19.3)
Moderate	12 (21.1)
Severe	34 (59.6)
Department	
General surgery	60 (68.2)
Gynecology	22 (25)
ENT	2 (2.3)
Others	4 (4.4)

*ASA* American Society of Anesthesiologist, *BMI* Body Mass Index, *Kg/m*^*2*^ Kilogram/squaremeter

The incidence of difficult laryngoscopy was 14.8%. The median (IQR) of thyromental distance in difficult laryngoscopy was 6.5 (6.3,8.0) cm compared with 7.5 (7.0,8.0) cm in easy laryngoscopy (*p*-value 0.03). The median (IQR) of sternomental distance in difficult laryngoscopy was 16.8 (15.2,18.0) cm compared with 16.0 (14.5,16.0) cm in easy laryngoscopy (*p*-value 0.05). The mean distance from skin to epiglottis was 12.2 ± 3.3 mm. Mean of distance from skin to epiglottis in difficult laryngoscopy was 12.5 ± 3.3 mm compared with 10.6 ± 2.9 mm in easy laryngoscopy (p-value 0.05) (Table 2). There was no statistically significant difference in BMI, severity of OSA, modified Mallampati score, thyromental distance, interincisor gap, upper lip bite test, or neck circumference between easy and difficult laryngoscopy.

**Table 2:** Variables comparing easy and difficult laryngoscopy. Data shown as Number(%) Mean ± SD or median (IQR)

Variables	Easy Laryngoscopy (***n*** = 75)	Difficult Laryngoscopy (***n*** = 13)	***P*** value
^b^Age (years)	38 (29,48)	48 (46,57)	0.08
^b^Body mass index (kg/m^2)^	44.0 (39.8, 50.8)	41.8 (37.4, 45.9)	0.19
^c^Snoring	72 (96)	12 (92.3)	0.48
^c^Polysomnography	48 (64)	9 (69.2)	1
^c^Apnea-Hyponea Index (AHI)			1
mild	9 (18.8)	2 (22.2)	
moderate	10 (20.8)	2 (22.2)	
severe	29 (60.4)	5 (55.6)	
^c^Modified Mallampati score			0.46
I	28 (37.3)	5 (38.5)	
II	24 (32)	6 (46.2)	
III	19 (25.3)	1 (7.7)	
IV	4 (5.3)	1 (7.7)	
^b^Thyromental distance (cm)	7.5 (7.0,8.0)	6.5 (6.3,8.0)	**0.03**
^b^Sternomental distance (cm)	16.8 (152,180)	16.0 (145,163)	**0.05**
^a^Interincisor gap (cm)	4.6 (0.7)	4.5 (0.8)	0.59
^a^Neck circumference (cm)	42.8 (4.3)	41.9 (3.8)	0.51
^c^Abnormal upper teeth	12 (16)	1 (7.7)	0.68
^c^Upper lip bite test			0.60
I	43 (57.3)	10 (76.9)	
II	29 (38.7)	3 (23.1)	
III	3 (4)	0 (0)	
^a^Distance from skin to epiglottis (mm)	10.5 (2.9)	12.5 (3.3)	**0.05**

^a^ Data are presented as mean (Standard Deviation)

^b^ Data are presented as median (Interquartile range)

^c^ Data are presented as number (Percentage)

Multivariate logistic regression associated with difficult laryngoscopy consisted of age more than 43 years, thyromental distance more than 68 mm, and distance from skin to epiglottis more than 13 mm (Table 3). Allocated weights to contribute to the predictive score are also shown in the table.

**Table 3:** Multivariate logistic regression of difficult laryngoscopy factors

Factors	Coefficient	Standard Error	*P*-value	95%CI	Allocated Weight
**Age > 43 years**	1.35	0.73	0.065	0.11 to 2.92	8
**Thyromental distance > 68 mm**	1.17	0.66	0.077	−0.19 to 2.35	7
**Distance from skin to epiglottis > 13 mm**	0.98	0.85	0.252	−0.70 to 2.65	6

*cm* centimeter, *mm* millimeter, *CI* Confident Interval

**Fig. 3:**
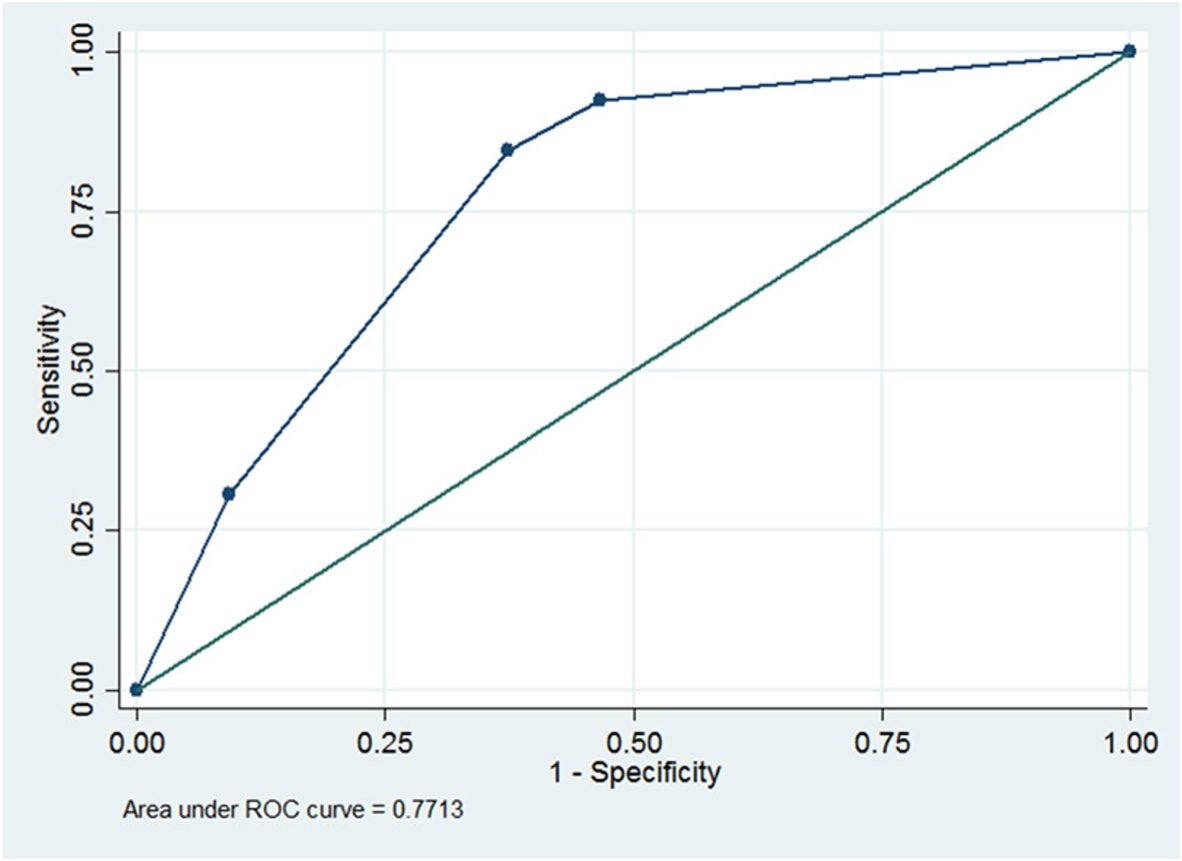
ROC Curve shown the probability of difficult laryngoscopy

The scores to predict difficult laryngoscopy was calculated as 8A + 7B + 6C. One point is given for A if age was more than 43 years old, 1 point is given for B if thyromental distance was less than 6.8 cm and 1 point is given for C if the distance from skin to epiglottis by ultrasonography was more than 13.0 cm. The scores, probability, odds and likelihood ratio are shown in Table 4. The maximum predicting score is 21, which indicates a probability of difficult laryngoscopy among our patients of 36.36%, odds 0.57, likelihood ratio 3.29 and area under the ROC curve of 0.78 in Fig. 3.

**Table 4:** The predicting scores of difficult laryngoscopy, odds and likelihood ratio

scores	Probability of difficult laryngoscopy	Odds	Likelihood Ratio
0–6	2.44	0.03	0.17
7–8	12.50	0.14	0.83
13–15	25.00	0.33	1.92
21	36.36	0.57	3.29

## Discussion

The prediction of difficulty airway has low sensitivity and specificity using standard technique [12]. The anesthesiologists always need to prepare for difficult ventilation and intubation especially in morbid patients. Kopanaki et al. [14] and Prakash et al. [15] found that sternomental distance was one of conventional parameters of airway assessment. We found that thyromental distance and sternomental distance predicted difficult laryngoscopy among obese Thai patients.

Gonzalez et al [9] found that the difficult tracheal intubation is more frequent in obese than lean patients (14.3% vs 3%; *p* = 0.03) similar to our study, which found that the incidence of difficult laryngoscopy was 14.8% among obese patients.

Airway ultrasound can assess airway anatomy prior to intubation. Many parameters are used to predict difficult intubation [12, 16, 17]. Recent studies have used ultrasonography-measured distance from skin to epiglottis [10] and pre-tracheal soft tissue at the level of vocal cords [11], and cut-off values of 27.5 mm and 28 mm respectively, are used to predict difficult laryngoscopy. However, Asian figures are likely to be different from Caucasians’. We found the distance between skin and epiglottis more than 13 mm can predict difficult laryngoscopy among obese Thai population.

We combined the conventional airway assessment and the parameter of the ultrasound airway assessment for giving more information before intubation. Thyromental distance, sternomental distance and the distance between the skin and epiglottis are the parameters that showed significant differences between easy and difficult laryngoscopy among obese Thai patients in a univariate logistic regression. Multivariate logistic regression revealed that the factors associated with difficult laryngoscopy consisted of age more than 43 years, thyromental distance more than 68 mm, and the distance from skin to epiglottis more than 13 mm. We calculated the score to predict difficult laryngoscopy using these parameters to indicate the probability and odds of difficult laryngoscopy, and the likelihood ratio for each value of the score. The discriminating ability of the model was shown by the area under the ROC curve of ROC 0.77.

## Conclusions

Age, thyromental distance and ultrasonography for the distance from skin to epiglottis can predict difficult laryngoscopy among obese Thai patients. The predicting scores showed the probability of difficult laryngoscopy.

### Limitations

First, this is a single center study collecting data. More multicenter studies in different Asian countries are needed to expand our conclusions to other patients suffering from obesity in the Asian population. Second, the proposed scoring system has limitations as it is not practical to use in day to day practice due to its complexity.

## Data Availability

The datasets used and/or analyzed during the current study available form the corresponding author on reasonable request.

## Abbreviations

ASA: American society of anesthesiology
BMI: Body mass index
CI: Confidence interval
DAS: Difficult airway society
IQR: Interquartile range
OSA: Obstructive sleep apnea
ROC: Receiver operating characteristic
SD: Standard deviation
